# Effects of Dietary Inclusion of Bilberry and Walnut Leaves Powder on the Digestive Performances and Health of Tetra SL Laying Hens

**DOI:** 10.3390/ani10050823

**Published:** 2020-05-09

**Authors:** Roua Gabriela Popescu, Sorina Nicoleta Voicu, Gratiela Gradisteanu Pircalabioru, Alina Ciceu, Sami Gharbia, Anca Hermenean, Sergiu Emil Georgescu, Tatiana Dumitra Panaite, Anca Dinischiotu

**Affiliations:** 1 Department of Biochemistry and Molecular Biology, University of Bucharest, 050095 Bucharest, Romania; roua.popescu@drd.unibuc.ro (R.G.P.); alina_ciceu@yahoo.com (A.C.); samithgh2@hotmail.com (S.G.); georgescu_se@yahoo.com (S.E.G.); anca.dinischiotu@bio.unibuc.ro (A.D.); 2 Department of Pharmacy, Faculty of Pharmacy, Titu Maiorescu University, 004051 Bucharest, Romania; 3 Department of Botany-Microbiology, The Research Institute of the University of Bucharest (ICUB), 76201 Bucharest, Romania; gratiela87@gmail.com; 4 Department of Experimental and Applied Biology, “Aurel Ardelean” Institute of Life Sciences, Vasile Goldis Western University of Arad, 310414 Arad, Romania; anca.hermenean@gmail.com; 5 National Research and Development Institute for Animal Biology (IBNA Balotești), 077015 Ilfov, Romania; tatiana_panaite@yahoo.com

**Keywords:** bilberry leaves, walnut leaves, laying hens, digestive enzyme activities, nutrition

## Abstract

**Simple Summary:**

In poultry, diet composition influences growth performance, egg production, as well as digestion. In this study, the effects of dietary additives obtained from bilberry and walnut leaves powder on the digestive performances of Tetra SL hens were evaluated by histologic and morphometric analyses of the intestinal mucosa as well as by the enzymatic activity measurements of alpha-amylase, invertase, maltase, and trypsin correlated with cecum microbiota.

**Abstract:**

The purpose of this study was to examine the effects of dietary inclusion of two additives at the final concentration of 0.5% bilberry (E1) and 1% walnut (E2) leaves powder in the basal diet on digestive health of hens. A total number of 90 Tetra SL hens were divided into two experimental groups (E1 and E2) and one control group (C) consisting of 30 hens each. After four weeks, 10 hens of each group were sacrificed and tissue samples and intestinal content were taken from the duodenum, jejunum, and cecum in order to perform histological, enzymatic, and microbiota analyses. In groups E1 and E2, the histological analysis showed a significant increase of villus height, resulting probably in increased absorption of nutrients in duodenum and jejunum. A decrease in the specific activity of alpha-amylase and trypsin in E1 and E2 for both duodenum and jejunum compared to the control one was also recorded. In addition, the maltase and invertase specific activity in duodenum increased, a tendency that was kept for maltase but not for invertase in jejunum. The cecal microbiota of E1 and E2 individuals was characterized by an increase of *Firmicutes* and *Lactobacilli* and a decrease of *Enterobacteriaceae*. In conclusion, our results indicate that bilberry and walnut leaves additives in feed may improve the health status of the poultry gastrointestinal tract.

## 1. Introduction

Tetra SL is a brown egg-laying hybrid widely used for its excellent internal and external egg quality as well as for efficient and long-term egg production. Animal performance improvement is the most important detail from an economic point of view, especially in the livestock industry. Composition of diets given to hens [1] influences growth performance, egg production, as well as digestion. In poultry nutrition, energy suppliers and proteins are the most important feed constituents after water. Cereals provide about 70% of energy, whereas other sources supply the rest. Recently, phytogenic feed additives received attention as alternatives to prebiotics, probiotics, and antibiotics in laying hen nutrition. Some herbal feed inclusions were shown to improve performance, immunity, and antioxidant status in laying hens [2]. Other ones did not affect body weight but improved egg production; weight; and quality regarding yolk color, cholesterol, and malondialdehyde compared to the control [3]. Furthermore, bilberry and walnut leaves powder included in the diet of laying hens increased the antioxidant properties of egg yolks [4] but did not affect other performance indicators.

The avian gastrointestinal tract is divided into nine discrete segments: the oral cavity, esophagus, crop, proventriculus, gizzard, small intestine, ceca, large intestine, and the cloaca. The small intestine is the major site for digestion and absorption of nutrients and influences the rates of energy intake, feeding behavior, and energy allocation [5]. The electrolytes together with digestive enzymes secreted by pancreas and intestinal glands and those produced by mucosal cells are responsible for the hydrolysis of macronutrients. The initial digestion of feed is carried out by pepsin and pancreatic proteases, peptidases, lipase, and amylase [6,7].

Currently, there are limited scientific data regarding the relationship between the nutritional quality of feed used in poultry farming and digestive enzymes [8,9]. Bird [10] determined the distribution of trypsin and amylase in different segments of duodenum in Leghorn chickens. Thus, the first three quarters of the duodenum have a content of 45% trypsin, with 55% in the last part, and for amylase, the content is 23% in the first part, most of the activity of amylase in duodenum being in the last third, almost from the point where the pancreatic ducts communicate with the duodenum [11].

Functional anatomical and histological characteristics of the avian gastrointestinal tract are critical to their feed conversion efficiency. To facilitate maximal absorption of dietary components, the intestinal mucosa is highly convoluted and specialized. The epithelium is folded into villi and the epithelial cells have apical microvilli, forming a brush border observed by optical microscopy. These infoldings increase the small intestinal surface area for absorption by about 600-fold, resulting in a higher capacity for nutrient absorption [12]. Intestinal morphology (villus height and crypt depth) changes in response to exogenous agents. Deeper crypts indicate faster tissue turnover as they contain progenitor cells. Intestinal mucins are high molecular weight glycoproteins secreted by goblet cells. In chickens, mucins are observed to be extensively expressed by goblet cells in the colon and small intestine [13].

Also, the gastrointestinal tract of poultry has a diverse and complex microbiota that plays a significant role in the digestive process and the absorption of nutrients, maintaining immune system development and pathogen exclusion, which are vital for improvement of digestion, health, and growth performance [14]. Poultry microbiota composition depends on many factors including the exposure to growth environmental, intestinal segment, and diet [15]. The esophagus, crop, and cloaca are considered semi-aerobic environments, allowing mixed communities of aerobes, micro-aerobes and facultative anaerobes, including members of the α, β, and γ-Proteobacteria. The internal sections of the gastrointestinal tract located between the crop and cloaca are dominated by obligate or facultative anaerobes, including members of the *Firmicutes* and *Proteobacteria* [16].

Obviously, the improvement of poultry growth performance depends on intestinal health and consequently on microbiome composition. An appropriate microbiota is favored by different vegetal additives. In this context, a method to improve animal performance is the use of vegetal feed additives, which have beneficial effects in livestock production as well as in health and nutrition of animals, which might arise from activation of feed intake and digestive secretions. These have also antimicrobial, antiviral, antioxidant, and immunomodulatory properties [17,18].

Starting from the fact that plants with high phenolic content have strong antioxidant power [19], and due to the few data regarding the use of plant leaves as additives in the diets of laying hens and their nutritional assessment, in this study, we have chosen to use bilberry and walnut leaves powder. *Vaccinium myrtillus* is a species of the genus *Vaccinium* from the family *Ericaceae* [20]. Fruit and aerial parts of plant are known as a natural source of food and drink due to their richness in nutritional and antioxidant compounds and can also be integrated into food supplements and pharmaceuticals for preventing urinary tract infections [21] and cerebral vascular accidents [22]. Bilberry has several effects such as prevention or even reversal in a considerable degree of age-related object memory decline of rats [23] and antioxidant [24,25], anti-inflammatory, anticancer, anti-neurodegenerative, and cardioprotective effects [26,27] due to their phenolic compounds, including proanthocyanidins, flavonoids, stilbenoids, phenolcarboxylic acid derivatives, and flavonol glycosides [20].

*Juglans regia L*. belonging to the *Juglandaceae* family is the most well-known member, representing an important species of deciduous trees. Walnut leaves have been considered as a beneficial source of health, with important amounts of phenolic compounds [28], and have been intensively used in traditional medicine [29] for the treatment of venous insufficiency and hemorrhoids and for their antidiarrheal, anthelmintic, astringent, keratolytic, antifungal, hypoglycemic, hypotensive, and sedative effects [29]. Also, they have high content in flavonoids, alkaloids, saponins, steroids, and tannins [30].

Previously, it has been shown that walnut leaves administration reduced the proliferation of *Clostridium perfringens* in chickens [31]. Also, Mousavi et al. [32] showed that supplementation of broiler chicken diet with a green husk of walnut powder improved the function of the immune system. The goal of this study was to examine the consequences of the dietary inclusion effects of additives obtained from bilberry and walnut leaves powder on the microbiota from cecum correlated with digestive performances of Tetra SL hens by histologic and morphometric analyses of the intestinal mucosa as well as the enzymatic activities measurements. As far as we know, this is one of the first studies regarding the correlation between dietary inclusion of some plant leaves and the health of poultry gastrointestinal tract.

## 2. Materials and Methods

### 2.1. Plant Material and Antioxidant Capacity

Phytochemical characterization of plant material and leaf samples of bilberry and walnut were obtained from local pharmacies (S.C. Stefmar productie S.R.L, Râmnicu Vâlcea, Romania). The leaf extracts of bilberry and walnut were prepared according to the method described by Coșarcă et al. [33]. A mass of 1 g of dried vegetable material was mixed with 40 mL distilled water and heated at 90 °C for 45 min with shaking. The suspension was then centrifuged at 2370× *g*, and the supernatant was stored at −20 °C until analysis was done [34].

The total polyphenol content in extracts was quantified according to the Folin–Ciocalteu method as described previously [35]. A sample of 50 µL was homogenized with 250 µL of 1/10 diluted Folin–Ciocalteu reagent and incubated for 1 min at room temperature. Then, a volume of 750 µL of 7.5% (w/v) Na_2_CO_3_ solution was added. The mixture was brought to a final volume of 5 mL and then incubated in the dark for 2 h at 25 °C. At the end, the optical density was measured at 760 nm using distilled water as a blank. The concentration of polyphenols was calculated using a gallic acid calibration curve. The results were expressed in milligrams of gallic acid (GAE) per gram of dried weight (d.w.) (mg GAE/g dw).

The antioxidant capacity of leaf extracts of bilberry and walnut was determined using the 2,2-diphenyl-1-picrylhydrazyl (DPPH) radical according to the method of Burits and Bucar [36]. Different leaf extract concentrations were mixed with 0.04% DPPH at a ratio of 1:100. After 30 min of incubation in the dark at room temperature, the absorbance of samples was measured at 517 nm using a FlexStation 3 multi-mode microplate reader (Molecular Devices LLC, San Jose, CA, USA) [37].

Oxygen radical absorbance capacity (ORAC) assay was performed according to the method of Davalos et al. [38]. A volume of 20 µL of extract or phosphate buffer (for blank) was incubated with 120 µL of 70 nM fluorescein for 15 min in darkness at 37 °C. The peroxyl radical was generated by adding 60 µL of 12 mM 2,2′-azobis (2-amidino-propane) dihydrochloride (AAPH), which was freshly prepared before each test. After 80 min of incubation in darkness at room temperature, the fluorescence intensity (FL) was recorded (excitation wavelength at 485 nm; emission wavelength at 520 nm) for 80 min at intervals of one minute using the FlexStation 3 multi-mode microplate reader (Molecular Devices LLC, San Jose, CA, USA). In parallel, a standard curve was prepared with Trolox (6-hydroxy-2,5,7,8-tetramethylcroman-2-carboxylic acid) at concentrations ranging 0–100 µM (0, 12.5, 25, 50, 75, and 100 µM) [37].

### 2.2. Hens and Experimental Treatments

A total of 90 Tetra SL laying hens (aged 32 weeks) was assigned into two experimental groups (E1 and E2) and one control group (C) with 30 birds each (ten replicates each; 3 birds/replicates; and a total of 30 cages) and housed in an experimental hall under controlled environmental conditions (temperature, humidity, and ventilation) in 3-tier batteries and 16 h/24 h light regimen. Feed and water were offered ad libitum during the experiment. The corn–soybean meal basal diet (2800 kcal/kg metabolizable energy (ME) and 17.8% crude protein (CP) was the same for all groups as described by Untea et al. [4], which contained 30% corn; 31.46% wheat; 4% gluten, 21.2% soybean meal; and 1.46% vegetable oil and other components per kg. Unlike the diet formulation for group C, the experimental diets included two different herbal feed additives as follows: 0.5% bilberry leaves (E1) and 1% walnut leaves (E2). Diet formulations were calculated in agreement with the feeding requirements of laying hens as given by National Research Council [39]. After four weeks, 10 hens of each group (randomly selected from each cage) were slaughtered with the approval (case no. 5148/10.08.2018) of the Ethical Committee of the National Research-Development Institute for Animal Nutrition and Biology, Balotești, Romania (Ethical Committee no. 52/30.07.2014).

Performance parameters regarding feed intake, feed conversion ratio, egg production, egg weight, and laying percentage were monitored on the experimental period. Herbal feed additives used did not influence the production performances of the birds except for the weight of the eggs, the results being presented previously by Untea et al. [4] as a part of this study. Also, the antioxidant stability of egg yolk was increased due to higher concentrations of lutein and zeaxanthin, that is very important for human nutrition [4].

### 2.3. Sample Collection for Analyses

The small intestine from each individual was collected, and intestinal content and tissue samples were taken from the duodenum and jejunum in order to perform enzymatic and histologic analyses. The intestinal contents from duodenum jejunum and cecum were also collected. The total protein extracts were obtained by homogenization of 1 g tissue in 10 mL phosphate buffer, pH 7.4. The suspensions were kept one hour at 4 °C and centrifuged at 9600× *g* for 10 min. The resulting supernatants were used for the determination of maltase and invertase activities. Also, the activities of alpha-amylase and trypsin were measured in the intestinal content, whereas the microbiological analyses were performed on the cecal content.

### 2.4. Light Microscopy Examination

The intestinal segments were immersed in 4% paraformaldehyde in phosphate buffered saline (PBS) solution and dehydrated in a graded series of ethanol. Finally, each specimen was embedded in paraffin and cut into 4-μm sections using a microtome. Hematoxylin and Eosin (H&E) (Merck, Darmstadt, Germany) stain was performed. Ten villi and crypts of Lieberkühn from duodenum and jejunum segments of each bird were measured using an optical microscope (Olympus BX43, Tokyo, Japan), a camera (30 XC Olympus, Tokyo, Japan), and image analysis software (Olympus Cell Sense Dimension, Tokyo, Japan). For measurement of villus height and widths of crypt, mucosa segments were randomly selected from each cross section. A total of 10 villus heights (measured from the tip of the villus to the villus–crypt junction) and the depth of 10 crypts (measured from the crypt–villus junction to the base of the crypt) from cross sections of each individual were analyzed.

### 2.5. Enzymatic Analysis

#### 2.5.1. Maltase Activity Assay

Maltase (EC 3.2.1.20) activity was determined using the Maltase assay kit instructions from My BioSource (San Diego, CA, USA). For each sample, an appropriate control was prepared. A volume of 25 µL from sample was mixed with 50 µL substrate (maltose) and incubated for 20 min at 37 °C. Then, the reaction was stopped by 25 µL stop solution and the mixture was centrifuged at 4000× *g* for 10 min. The supernatant was mixed with 200 μL of chromogenic agent, homogenized, and incubated for 15 min at 37 °C. The absorbance was measured using the Flex Station 3 Multi-Mode Microplate Reader (Molecular Devices LLC, San Jose, CA, USA) automatic plate reader at 505 nm. The enzymatic activity was calculated using a calibration curve with glucose. The results obtained were expressed in U/mg of protein.

#### 2.5.2. Invertase Activity Assay 

The enzymatic activity of invertase (EC 3.2.1.26) was evaluated according to the Invertase assay kit instruction (code MAK 118; Sigma-Aldrich, Darmstadt, Germany). Briefly, over a volume of 40 µL from each sample was added 5 µL of sucrose solution in each well. Then, the mixture was incubated for 20 min at room temperature. After this interval, a volume of 90 µL reaction mixture containing enzyme and dye reagent was added and incubated for 20 min in dark. The absorbance was measured at the FlexStation 3 Multi-Mode Microplate Reader (Molecular Devices LLC, San Jose, CA, USA) automatic plate reader at 570 nm. As a standard, a 100 µM glucose solution was used. The results obtained were expressed in U/mg of protein.

#### 2.5.3. Alpha-Amylase Assay

Alpha-Amylase (EC 3.2.1.1) activity was determined according to Bernfeld [40]. The amount of reducing sugars released from soluble starch was measured using an alkaline 3, 5-dinitrosalicylic acid (DNS) reagent. Therefore, 100 µL of 20 mM Na_2_HPO_4_–NaH_2_PO_4_ (pH 6.9) buffer and 100 µL of 1% soluble starch solution were mixed with 5 µL of sample and incubated for 10 min at 25 °C. The reaction was stopped by addition of 200 µL of DNS reagent. The reaction mixture was heated at 100 °C for 4 min. The reducing groups were quantified at 546 nm with a FlexStation 3 Multi-Mode microplate reader. One unit of activity represented the amount of enzyme that released one µmole of maltose in one minute at 25 °C. Enzyme activity was expressed as specific activity (units per gram of protein).

#### 2.5.4. Trypsin Assay

Trypsin (EC 3.4.21.4) activity was determined according to the method described by Hummel [41] using *N-p*-Tosyl-L-arginine methyl ester hydrochloride (TAME) as substrate. The change in absorbance at 247 nm was measured for 10 min at 25 °C on a FlexStation 3 Multi-Mode microplate reader (Molecular Devices LLC, San Jose, CA, USA). The reaction mixture contained 216.6 µL of 50 mM Tris-HCl (pH 8.0) buffer, 30 µL of 10 mM TAME, and 8.66 µL of sample. One unit of trypsin activity was defined as the amount of enzyme hydrolyzing one micromole of TAME in one minute at 25 °C. Enzyme activity was expressed as specific activity (units per gram of protein).

### 2.6. Protein Determination

Protein concentration was determined by Bradford method [42] using bovine serum albumin as standard.

### 2.7. Microbiota Characterization

The Real-Time Quantitative Reverse Transcription PCR (qRT-PCR) technique was used to analyze the changes induced by the different diets at the microbiota level. Microbial DNA extraction was performed using a commercial kit (AllPrep PowerViral DNA/RNA Kit, Qiagen, Hilden, Germany). Briefly, 250 mg of cecal content was subjected to treatment with cell lysis matrix (PowerBead Tubes, Glass 0.1 mm, Qiagen, Hilden, Germany) as well as enzymatic digestion that resulted in nucleic acid isolation. Nucleic acids were subsequently subjected to purification (based on MB Spin Columns, according to the manufacturer instructions). Microbial DNA concentration and purity were spectrophotometrically quantified. The concentration of all DNA samples was adjusted to 3 ng/µL in DNAse and RNAse free water. The relative abundance of microorganisms in cecal DNA was measured by qRT-PCR on Applied Biosystem ViiA7. The total number of bacteria in the samples was quantified using universal primers for 16S rRNA. qRT-PCR reactions were performed using SYBR Green Master mix (Applied Biosystems) and specific primers for different taxa (all primers were selected from literature, and their sequences are shown in Table 1) [43,44,45,46]. Each PCR reaction included 200 nM forward and reverse primer, 9 ng DNA, and 2xSYBR Green Master Mix (Applied Biosystems, Foster City, CA, USA). The samples were incubated at 95 °C for 5 min and then amplified by 40 cycles of 95 °C for 10 s, 60 °C for 30 s, and 72 °C for 1 s.

### 2.8. Statistical Analysis

Statistical analysis of data was performed with GraphPad Prism software (Version 6, GraphPad, San Diego, CA, USA) using one-way ANOVA, followed by Bonferroni’s post hoc test. For histology, enzymology, and microbiota experiments, the number of replicates was n = 10. All values are expressed as mean ± standard deviation (SD) of three replicates, and the data were considered statistically significant only if the *p*-values were less than 0.05.

## 3. Results

### 3.1. Composition Analysis of Vegetable Additives

The antioxidant potential of each leaf extract was estimated using three methods: total polyphenols content measurement, DPPH free-radical scavenging activity, and ORAC assay (Table 2).

According to our data, the total polyphenol content is almost double in the bilberry leaves compared with the walnut leaves (Table 2). 

Also, the extract from bilberry leaves exhibited an 84.8% DPPH scavenging activity, confirming that this feed additive acts as a free radical scavenger. At the same time, it was an effective scavenger of the 2,2′-azino-bis (3-ethylbenzothiazoline-6-sulphonic acid) (ABTS) radical, according to the ORAC test. The antioxidant activity of walnut leaves extract was lower, presenting a 57.59% DPPH scavenging activity and almost half capacity to counteract the ABTS radical in comparison with bilberry leaves.

### 3.2. Histology of the Duodenum

**Figure 1:**
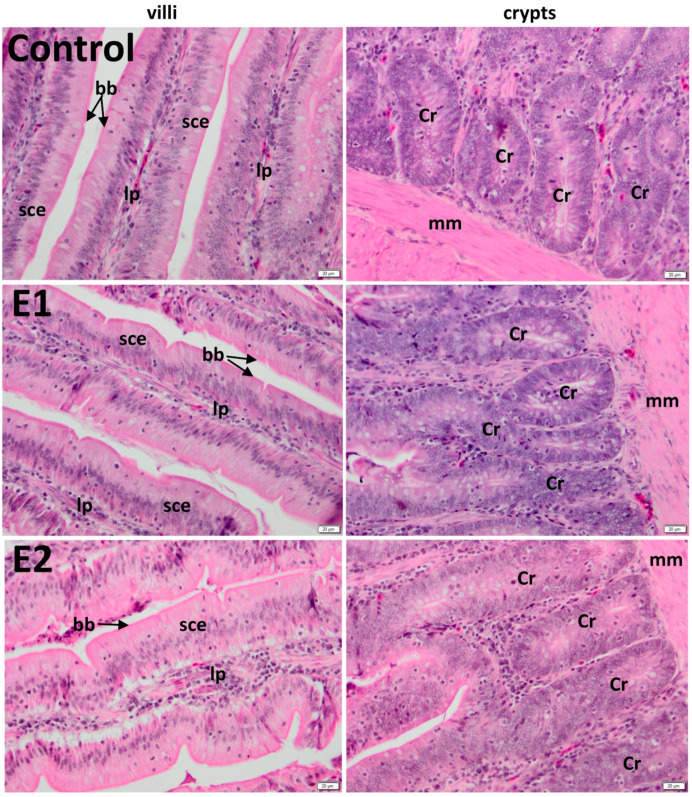
Effect of bilberry and walnut leaves supplementation diet on duodenum morphology of laying hens: The basal diet served as the control, and different levels of herbal feed additives were supplemented to the basal diet as follows: 0.5% cranberry leaves (E1) and 1% walnut leaves (E2). Sce: simple columnar epithelium; lp: lamina propria; cr: crypt; mm: muscularis mucosa. bb: brush border; H&E (Hematoxylin and Eosin) stain; (n = 10); n: number of replicates.

The duodenal villi were lined by a simple columnar epithelium, followed by a *lamina propria* made by connective tissue rich in vascular network, which absorbs the digestive products, and a *muscularis mucosa*, which underlies the base of crypts. The epithelium has a normal aspect, consisting of absorptive cells, called enterocytes, and individual goblet cells, which secrete mucin, for protection and lubrication of the intestinal contents. Brunner’s glands were not present in duodenum (Figure 1).

### 3.3. Histology of the Jejunum

**Figure 2:**
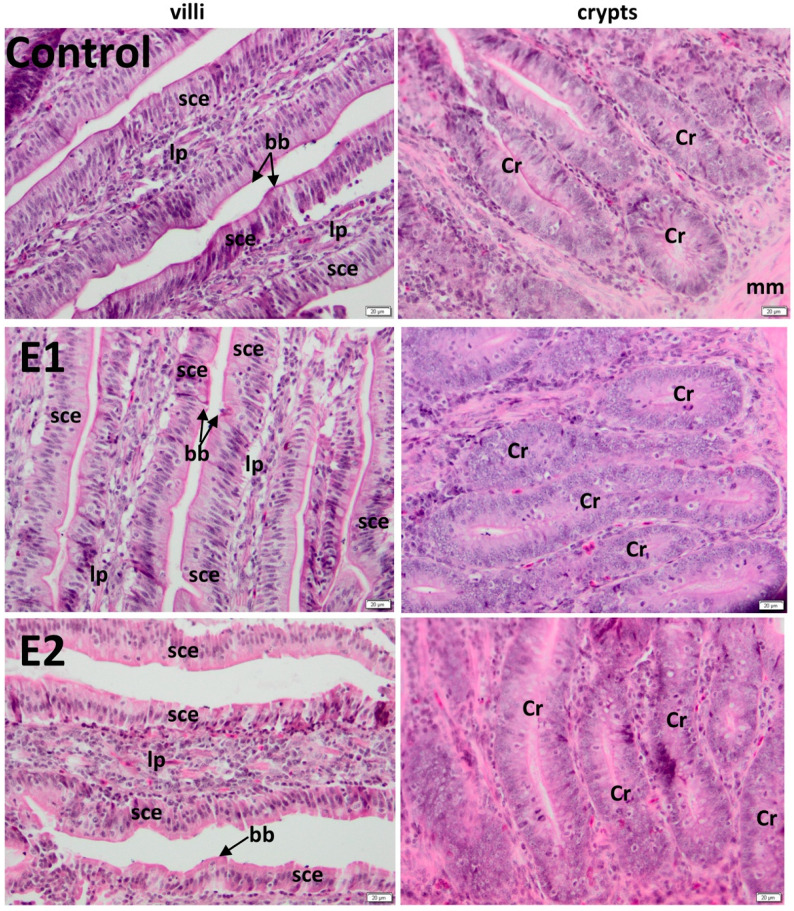
Effect of bilberry and walnut leaves supplementation diet on jejunum morphology of laying hens: The basal diet served as a control, and different levels of herbal feed additives were supplemented to the basal diet as follows: 0.5% cranberry leaves (E1) and 1% walnut leaves (E2). Sce: simple columnar epithelium; lp: lamina propria; Cr: crypt; mm: muscularis mucosa. bb: brush border; H&E (Hematoxylin and Eosin) stain; (n = 10); n: number of replicates.

Histology of the jejunum has a normal aspect (Figure 2).

The average lengths of villi of duodenum were 891.55 μm (control), 1158.84 μm (E1), and 1263.44 μm (E2), whereas the average widths of crypts of duodenum were 189.94 μm (control), 189.49 μm (E1), and 189.46 μm (E2) (Table 3).

Also, the average lengths of villi of jejunum were 905.98 μm (control), 1258.04 μm (bilberry leaves E1), and 1248.7 μm (walnut leaves E2). Moreover, the average widths of the crypts of jejunum were 209.51 μm (control), 210.26 μm (E1), and 211.39 μm (E2) (Table 3). Villus height (Table 3) was significantly higher for the experimental groups compared to control (*p* < 0.001).

### 3.4. Intestinal Enzymes Activities

In duodenum, the activity of maltase (Table 4) increased insignificantly by 157% for E1 by 264% for E2 compared to control. In jejunum, the same enzymatic activity decreased by 86% in the case of the E1 group, while for E2, it was increased by 330% (Table 4) compared to control. Regarding the specific activity of invertase in duodenum, it was unmodified in group E1 whereas, in group E2, it increased significantly by almost 11.61% compared to the control (Table 4). In contrast, the administration of basal diet enriched with 1% walnut leaves decreased significantly by 8.89% in jejunum. The basal diet supplemented with 0.5% bilberry leaves (E1) determined an increase of invertase specific activity at the jejunum level compared to the control level.

Our experimental data revealed a decrease in the specific activity of alpha-amylase in experimental groups for both duodenum and jejunum compared to the control. Furthermore, the enzymatic activity of trypsin from both intestinal segments was insignificantly decreased and below the control group level in all experimental groups.

### 3.5. Intestinal Microbiota

The modification of the diet recipe caused the appearance of changes in the intestinal microbiota. These were evident at the phylum level as well as at the bacterial population level. In general, *Firmicutes* and *Bacteroidetes* are the most abundant phyla, followed by *Proteobacteria*, *Actinobacteria*, *Verrucomicrobia*, and *Fusobacteria* [47]. The major phyla that make up the gut microbiota (*Bacteroidetes* and *Firmicutes*) have been analyzed. The administration of the commercial recipe enriched with bilberry leaves (E1) or walnut leaves (E2) led to a moderate decrease in the level of *Bacteroidetes* and an increase in the abundance of *Firmicutes* (Table 5).

Generally, the personalization of diet formulas aims to induce beneficial modifications of the microbiota by increasing the number of bacteria with probiotic potential (e.g., lactobacilli) and by decreasing the level of potentially pathogenic bacteria (pathobionts) such as *Enterobacteriaceae*. In this context, we quantified the abundance of lactobacilli and members of the *Enterobacteriaceae* family in isolated cecal samples from hens fed with different dietary formulas. For both analyzed groups, a significant decrease of the level of *Enterobacteriaceae* was observed as well as a statistically significant increase of the level of lactobacilli (Table 5).

## 4. Discussion

The poultry industry is one of the most dynamic animal industries. Feed efficiency and high performance of birds as well as the quality of eggs are the crucial goals in poultry production. In this context, the quality of diet along with environmental conditions and health of birds need to be considered to achieve these goals [48].

Nowadays, different herbal additives have been investigated for antioxidant, antimicrobial, and anti-inflammatory activities; growth-promoting effects; and egg quality. The beneficial impact of herbal additives could be due to the polyphenolic composition, which influences sugar digestion and absorption of nutrients in the small intestine [49]. Higher antioxidant protection of bilberry leaves compared to walnut ones could be conferred by the raised polyphenol content that can neutralize free radicals and can inhibit the propagation of free-radical reactions [50,51].

In the current study, histological evaluation showed that bilberry and walnut leaves powder supplementation of diets have exerted beneficial effects in the duodenum and the jejunum morphology, materialized in significant increases of villus height, resulting probably in increased adsorption of nutrients.

This hypertrophy of villi and, by default, of their epithelial cells resulted in an increased surface area and capacity of absorption [52], corresponding to raised activities of intestinal enzymes [53,54] probably due to feed supplementation with bilberry and walnut leaves powder. Previously, it has been stated that villi height and crypt depth ratio in the small intestine has a direct influence on the absorptive function [55]. These have been observed in chickens after the administration of zeolite [56], L-glutamine [57], and clinoptilolite [55].

In poultry, the activity of digestive enzymes located in the brush border membrane of enterocytes plays a significant role in the physiological processes occurring in the digestive tract that depend on nutritional feeding and characteristics of diet [5]. Previously, it has been shown that activities of digestive enzymes are affected by the amount, composition, and regime of food intake during the growing phase [11]. Several studies have shown that the activities of proteases in the intestinal juice are modified according to the amount of protein in the diet, while the activities of amylase and lipase depend on the content of food in carbohydrates and lipids as substrates for their activity [7].

According to our data, a strong negative correlation between the total polyphenol content and a decreased alpha-amylase and trypsin activities both in duodenum and jejunum was observed (Table 2).

The alpha-amylase catalyzes hydrolysis of the internal α-1, 4-glycosidic linkages in starch, generating glucose and maltose. In our study, the activity of alpha-amylase is decreased in both experimental groups E1 and E2 for duodenum and jejunum compared to control. Considering that amylase activity is diminished, probably oligosaccharides escaped digestion in the small intestine and reached the cecum, where they were fermented by microbiota producing short chain fatty acids [58] and other aminated compounds with beneficial impact on hens. There are three main short chain fatty acids, namely acetate, propionate, and butyrate, that represent signaling molecules with epigenetic impact [59].

Trypsin is responsible for protein and peptides degradation into amino acids. The peptides hydrolysis stage is important in protein absorption because, although these are smaller in size compared to proteins, they are still too large to be absorbed by the small intestine mucosa [60]. Our results show that trypsin activities from duodenum and jejunum were insignificantly decreased and below the control level in all experimental groups E1 and E2, probably due to covalent attachment of the phenolic compounds from feed additives to reactive nucleophilic sites of the enzyme, affecting its three-dimensional conformation and the active site. As a result, the rate of the catalyzed reaction decreased significantly [61].

Maltase and invertase are key enzymes of carbohydrate digestion; therefore, the relationship between cecum microbiota and small intestine enzyme activity could be due to glucose and fructose presence in the gastrointestinal tract, which could favor the *Lactobacilli* strains’ presence as previously has been proved [62,63].

It has been shown that partial digests of protein in the culture medium was essential for growth of some lactic bacteria [64]. Also, peptides resulting in the small intestine due to trypsin activity could favor the relative abundance of *Firmicutes* [65].

An interesting observation is that, in hens from the E1 and E2 groups, duodenal invertase activity increased compared to control, whereas in the case of jejunum, this activity was higher for E1 and decreased for E2. Invertase is an enzyme with two active centers [66], one that catalyzes the hydrolysis of sucrose and the other involved in the hydrolysis of both maltose and other alpha-glucosides.

Probably due to the increased activity of invertase in the E2 group in duodenum, a lower amount of sucrose was present in jejunum, and this could explain the decreased invertase activity (Table 4) and the increased maltose hydrolysis (Table 4).

For *Aves* species, the digestion starts at the oral cavity and ends at the cloacal level, crossing several “stations” represented by a series of organs involved in the process [67]. The cecum is presented in the form of two extensions at the intersection of the small intestine with the large intestine [68] and is specialized in fiber digestion, nitrogen recycling through urine and osmotic regulation, and water resorption [69]. At the same time, certain types of B vitamins are released (thiamine, riboflavin, niacin, pantothenic acid, pyridoxine, biotin, folic acid, and vitamin B_12_). Dietary fiber components are not digested by endogenous digestive enzymes and consequently are substrates for bacterial fermentation in the distal part of the gut [70].

Our results indicate that group E2 (commercial recipe + 1% walnut leaves) has the highest probiotic potential, with this type of diet leading to a 5-fold increase in the level of lactobacilli and a significant decrease in *Enterobacteriaceae* compared to the control group. Moreover, probably higher concentrations of short chain fatty acids produced in the small intestine decreased the *Enterobacteriaceae* population [71], which is negatively correlated with the *lactobacilli* population (Table 5). Similarly, Leusnik et al. [72] showed that dietary supplementation with bilberry extract (80 mg/kg of feed) significantly decreased the size of *Enterococcus* spp. populations in broilers on day 28 of the experiment.

## 5. Conclusions

Our results indicate that basal diets enriched with bilberry and walnut leaves powder might change positively the microbiota of hens by modulating several digestive enzymes that favor the development of lactobacilli and decrease Enterobacteriaceae. As a result, we could conclude that supplementation of basal feed with herbal additives might increase the health status of poultry.

## Figures and Tables

**Table 1:** Primer sequences used for microbiota characterization.

Taxonomic Target	Primer Sequence
*Eubacteria*	UniF340 ACT CCT ACG GGA GGC AGC AGT UniR514 ATT ACC GCG GCT GCT GGC
*Lactobacilli*	LabF362 ACG AGT AGG GAA ATC TTC CA LabR677 CAC CGC TAC ACA TGG AG
*Enterobacteriaceae*	Uni515F GTG CCA GCM GCC GCG GTAA Ent826R GCC TCA AGG GCA CAA CCT CCA AG
*Bacteroidetes*	Bact934F GGAACATGTGGTTTAATTCGATGAT Bact1060R AGCTGACGACAACCATGCAG
*Firmicutes*	Firm934F GGAGCATGTGGTTTAATTCGAAGCA Firm 1060R AGCTGACGACAACCATGCAC

**Table 2:** Phytochemical characterization of plant material.

Parameters	Bilberry Leaves		Walnut Leaves	
	Average	SD	Average	SD
TPC (µg GAE/g d.w.)	392.644	±12.531	192.025	±10
DPPH (% inhibition)	84.807	±1.24	57.589	±3.17
ORAC (µmol Trolox/g d.w.)	328.908	±7.21	175.700	±4.5

TPC: Total polyphenolic content; GAE: Gallic Acid Equivalents; d.w: dry weight; DPPH: the antioxidant capacity using the 2,2-diphenyl-1-picrylhydrazyl radical; ORAC: Oxygen radical absorbance capacity value. All data are reported as mean plus or minus standard deviation (SD), (n = 3).

**Table 3:** Measurements of the villi length and widths of crypt for the control and experimental groups.

	Duodenum		Jejunum	
Group	Villus (µm)	Crypt (µm)	Villus (µm)	Crypt (µm)
C	891.55 ± 15	189.94 ± 11	905.98 ± 27	209.51 ± 27
E1	1158.84 ± 20 ***	189.49 ± 11 ns	1258.04 ± 21 ***	210.26 ± 44 ns
E2	1263.44 ± 23 ***	189.46 ± 14 ns	1248.7 ± 18 ***	211.39 ± 37 ns

C group: basal diet/control group; E1 group: basal diet with 0.5% bilberry leaves and E2 group: basal diet with 1% walnut leaves. All data are reported as mean values ± standard deviation (SD) and statistical significance, where ns *p* > 0.05, *** *p* ≤ 0.001; (n = 10); n: number of replicates.

**Table 4:** Influence of dietary source on enzymatic specific activity (U/mg protein) of maltase, invertase, alpha-amylase, and trypsin of duodenum and jejunum of laying hens.

Intestinal Segment	Group	Maltase (U/mg)	Invertase (U/mg)	Alpha-Amylase (U/mg)	Trypsin (U/mg)
Duodenum	C	443.87 ± 92.65	8.68 ± 3.49	208.69 ± 23.63	131.33 ± 52.29
	E1	600.63 ± 306.37 ns	7.98 ± 7.37 ns	54.89 ± 10.91 *	52.43 ± 8.95 ***
	E2	707.39 ± 245.02 ns	20.29 ± 6.68 ***	63.43 ± 23.74 ns	42.95 ± 1.75 ***
Jejunum	C	559.18 ± 25.19	25.69 ± 2.22	18.14 ± 5.5	98.01 ± 8.17
	E1	473.00 ± 64.34 ns	32.23 ± 5.96 ns	14.06 ± 3.88 **	61.68 ± 28.34 *
	E2	889.09 ± 79.07 ns	16.80 ± 3.43 ***	15.37 ± 3.85 ns	71.24 ± 16.66 *

The basal diet served as a control (C), and different levels of herbal feed additives were supplemented to the basal diet as follows: 0.5% bilberry leaves (E1) and 1% walnut leaves (E2). All data are reported as mean values ± standard deviation (SD) and statistical significance, where ns: *p* > 0.05; * *p* ≤ 0.05; ** *p* ≤ 0.01; *** *p* ≤ 0.001; (n = 10); n: number of replicates.

**Table 5:** The relative abundance of *Firmicutes, Bacteroidetes, Lactobacillus* sp, and *Enterobacteriaceae* phyla as determined by The Real-Time Quantitative Reverse Transcription PCR (qRT-PCR): Eubacteria 16S rRNA was used for normalization.

Group	*Bacteroidetes*	*Firmicutes*	*Lactobacillus* sp.	*Enterobacteriaceae*
C	1.15 ± 0.39	0.90 ± 0.41	1.11 ± 0.59	0.73 ± 0.33
E1	1.09 ± 0.21 ns	1.45 ± 0.52 ns	1.26 ± 1.57 **	0.005 ± 0.01 **
E2	0.97 ± 0.17 ns	1.56 ± 0.35 ns	4.55 ± 1.34 **	0.058 ± 0.05 **

C: control group; E1 group: basal diet with 0.5% bilberry leaves and E2 group: basal diet with 1% walnut leaves. All data are reported as mean values ± standard deviation (SD) and statistical significance, where ns: *p* > 0.05, ** *p* ≤ 0.01; (n = 10); n: number of replicates.
